# Muscle Energetics During Explosive Activities and Potential Effects of Nutrition and Training

**DOI:** 10.1007/s40279-014-0256-9

**Published:** 2014-10-30

**Authors:** Kent Sahlin

**Affiliations:** Åstrand Laboratory of Work Physiology, GIH, The Swedish School of Sport and Health Sciences, Lidingövägen 1, Box 5626, 11486 Stockholm, Sweden

## Abstract

The high-energy demand during high-intensity exercise (HIE) necessitates that anaerobic processes cover an extensive part of the adenosine triphosphate (ATP) requirement. Anaerobic energy release results in depletion of phosphocreatine (PCr) and accumulation of lactic acid, which set an upper limit of anaerobic ATP production and thus HIE performance. This report focuses on the effects of training and ergogenic supplements on muscle energetics and HIE performance. Anaerobic capacity (i.e. the amount of ATP that can be produced) is determined by the muscle content of PCr, the buffer capacity and the volume of the contracting muscle mass. HIE training can increase buffer capacity and the contracting muscle mass but has no effect on the concentration of PCr. Dietary supplementation with creatine (Cr), bicarbonate, or beta-alanine has a documented ergogenic effect. Dietary supplementation with Cr increases muscle Cr and PCr and enhances performance, especially during repeated short periods of HIE. The ergogenic effect of Cr is related to an increase in temporal and spatial buffering of ATP and to increased muscle buffer capacity. Bicarbonate loading increases extracellular buffering and can improve performance during HIE by facilitating lactic acid removal from the contracting muscle. Supplementation with beta-alanine increases the content of muscle carnosine, which is an endogenous intracellular buffer. It is clear that performance during HIE can be improved by interventions that increase the capacity of anaerobic ATP production, suggesting that energetic constraints set a limit for performance during HIE.

## Introduction

The energy demand increases in proportion to exercise intensity, and energy supply is often a critical factor for performance. During high-intensity exercise (HIE), anaerobic processes provide an extensive part of adenosine triphosphate (ATP) regeneration and accumulation of metabolic end-products (i.e. H^+^ and inorganic phosphate [Pi]) may disturb cellular homeostasis and muscle contraction. In this review, HIE is defined as exercise at an intensity exceeding that corresponding to maximal oxygen uptake (*V*O_2max_). Anaerobic processes dominate during all-out exercise of a duration less than 1–2 min [1], but also have an essential role at submaximal work rates (70–90 % of *V*O_2max_), where muscle lactate may reach critically high levels. Improvements in anaerobic capacity with training or ergogenic supplements will improve performance primarily during short-term (<7 min) maximal exercise, when anaerobic energy release is an essential factor. Many sports include explosive activities where HIE occurs during a sustained period (sprint and middle distance) or during short repeated bursts with intervening periods of low-intensity exercise (e.g. team-sports such as soccer, handball, and basketball). Aerobic energy release is mainly limited by the rate at which ATP can be produced (i.e. aerobic power or *V*O_2max)_. In contrast, anaerobic processes have a high power and are instead limited by the amount of ATP that can be produced (capacity). The capacity of the anaerobic processes is determined by the muscle store of high-energy phosphates and the maximal amount of lactate/protons that can be produced. By measurements of the maximal accumulated oxygen deficit, anaerobic capacity has been estimated to 52–90 ml O_2_, which corresponds to the energy demand of 1–2 min exercise at *V*O_2max_ [2].

Basic biochemistry tells us that anaerobic glycolysis leads to an equal production of lactate and hydrogen ions [3]. Most of the released hydrogen ions will be buffered, and only a small part (~0.001 %) will be free in the cytosol, resulting in a decrease in muscle pH. The decrease in muscle pH will interfere with biochemical and physiological processes and has for a long time been considered a factor in fatigue [4]. Buffering of protons will attenuate the change in pH and is the first line of defense against potential negative effects of lactic acid formation. Some of the formed lactate will be released to blood or be oxidized within the muscle. The efflux of lactate from the muscle [5] and oxidation of lactate will be accompanied by a similar amount of H^+^ removal. Both increases in muscle buffer capacity and enhanced removal of lactate/protons will increase the capacity of glycolytic ATP production.

Performance during sprint exercise is, to a large extent, determined by anthropometric and biomechanical factors. However, after a few seconds of maximal activity there is already a decline in maximal performance, indicating fatigue. The mechanism(s) of fatigue is a fundamental issue of exercise physiology and has been a topic of much debate. There is consensus that the mechanism of fatigue is multifactorial, dependent on both intrinsic factors (neuromuscular and central factors) and external factors (duration and type of exercise, environmental factors). An in-depth discussion of fatigue is outside the scope of this article, and the reader is referred to comprehensive reviews [4, 6, 7]. Performance during HIE can be improved with interventions (training and/or nutrition) that improve cellular energy production and/or attenuate the acidic state in the muscle. This supports the view that metabolic factors limit HIE performance and that energy deficiency and/or acidosis (related to lactate accumulation) are involved in the fatigue process.

## Relative Importance of Phosphocreatine (PCr) and Anaerobic Glycolysis to Anaerobic Energy Production

The muscle store of phosphocreatine (PCr) can be depleted almost completely during exhaustive exercise, providing an equimolar amount of ATP (about 70 mmol per kg dry muscle [dm]) in humans. Anaerobic glycolysis (i.e. glycogen to lactate) gives 1.5 mmol ATP per mmol of lactate. During exhaustive cycling at *V*O_2max_, muscle lactate can increase from 5 to 113 mmol/kg dm [8] corresponding to 162 mmol ATP/kg dm. Some of the produced lactate will be co-transported with H^+^ to the extracellular fluid, thus decreasing the intracellular acid load and enabling further glycolysis. Lactate efflux is dependent on muscle blood flow (capillary density and degree of restricting muscle contraction) [9], the amount of lactate transport proteins [10], the extracellular buffer capacity [11], and the extracellular lactate concentration [9]. Increasing the efflux of lactate will enhance glycolytic ATP production. The amount of lactate transported to blood has been estimated to be about 17 % of total lactate production during cycling at *V*O_2max_ [12] but will be lower during exercise of shorter durations. The contribution of anaerobic glycolysis (i.e. lactate formation) to anaerobic ATP production during cycling at *V*O_2max_ is thus 195 mmol/kg dm (162 + 33 mmol/kg dm) or 74 % (195/(195 + 70)) of total anaerobic ATP production, with the remaining part covered by PCr depletion. Although there is extensive anaerobic energy utilization during this type of exercise (cycling at *V*O_2max_), with concomitant depletion of PCr stores and high lactate levels in muscle and blood, the major part (84 %) of the energy demand is covered by aerobic processes [13].

The proportion of ATP derived from PCr utilization and anaerobic glycolysis is dependent on the duration and intensity of exercise. The maximal rate of ATP supply from PCr is higher than that from glycolysis and, during the first 3 s of contraction, PCr breakdown contributes to 70 % of the ATP formation [14]. The rate of PCr breakdown declines after a few seconds of maximal contraction, after which glycolysis increases in importance. There is a complex metabolic interaction between both aerobic and anaerobic processes and between PCr utilization and glycolysis. PCr utilization is coupled to increased concentration of Pi, which is a limited substrate for the flux-generating step of glycolysis, i.e. glycogen phosphorylase [15]. The increase in Pi will promote glycolysis and could explain the shift in ATP provision from PCr to glycolysis. Alternately, the reduced rate of PCr utilization may be a consequence of reduced muscle content of PCr and kinetic constraints of the creatine kinase reaction, where the reduced PCr/Cr ratio will reduce the maximal rate of PCr breakdown [16]. A reduction in PCr may therefore reduce anaerobic power and contribute to fatigue even before complete depletion of the PCr store.

The proportion of anaerobic ATP production covered by PCr will be high at the onset of exercise, whereas that of glycolysis will dominate after about 6 s of exercise [17]. PCr is rapidly resynthesized during recovery (half of the depletion restored after ~30 s [18]), whereas removal of muscle lactate is slower (half of the accumulated lactate removed after ~10 min [8]). Therefore, during interval exercise with short recovery periods, PCr utilization will be the dominating process of anaerobic ATP production [17, 19].

To sum up, the relative importance of PCr utilization and anaerobic glycolysis for anaerobic ATP production will depend on the duration, intensity, and type of exercise. During sustained HIE with a duration exceeding 6 s, anaerobic glycolysis will dominate, whereas at shorter durations and especially during interval exercise with short recovery periods, PCr will be the main source of anaerobic ATP.

## Improving Energy State with Training

Several studies have shown that muscle contents of ATP and PCr (per g of muscle) are not changed by prolonged training (for references see Saltin and Gollnick [20]). However, increasing the contracting muscle mass by resistance or sprint training will increase the total amount of ATP-PCr that can be used during exercise. An increased contracting muscle mass will also increase the distribution volume of lactate and thus enhance the amount of ATP that can be produced through anaerobic glycolysis. Training-induced hypertrophy will thus increase anaerobic capacity and have the potential to improve performance during HIE. Sprint training may alter the neuromuscular control by modifying the relative sequencing of muscle activation and increasing the recruitment or firing frequency of fast-twitch motor units [21]. This is in analogy with resistance training, where the initial increase in muscle strength is attributed to neural factors and not to hypertrophy. Sprint training-induced neural adaptations may, besides the beneficial effects on contraction speed and muscle power, increase the working muscle mass and thus anaerobic capacity. However, knowledge of how sprint training affects the neural system is limited due to the technical difficulties involved [21].

Resynthesis of PCr can only occur during aerobic conditions with ATP produced through oxidative phosphorylation [18], and the rate of PCr resynthesis is therefore dependent on muscle oxidative capacity. Training protocols that stimulate mitochondrial biogenesis will enhance the rate of PCr resynthesis [22] and is expected to improve performance during interval exercise with short rest/exercise periods (e.g. team sports), where PCr utilization is the dominating source of ATP during the working phase.

## Improving Buffer Capacity with Training

Lactate production is limited by the extent of the lactate-induced acidosis, i.e. the decrease in muscle pH. Muscle pH in humans may decrease from 7.0 at rest down to 6.4–6.5 at fatigue [23]. Buffering of protons will attenuate changes in pH at a certain proton load, and increases in muscle buffer capacity (induced by training or nutrition) will increase the amount of lactate that can be accumulated in the muscle. Several methods are available to determine muscle buffer capacity but, due to the complexity, none are free from criticism. Most studies have determined buffer capacity in vitro by titration, which will not include transmembrane transport of acid-base substances or dynamic buffering by biochemical processes. Measurements of buffer capacity with magnetic resonance spectroscopy are based on the false assumption of negligible lactate production during the initial phase of contraction [24]. Although absolute values of muscle buffer capacity may be uncertain, several studies have shown that high-intensity training can improve muscle buffer capacity in both untrained [25–27] and endurance-trained subjects [26, 28]. High-altitude training has also been shown to improve muscle buffer capacity [29]. Muscle buffer capacity is determined by several components, the major ones of which are PCr–Pi, protein, bicarbonate–CO_2_, and carnosine [23]. It is not clear which components of buffer capacity are altered by training.

## Creatine Supplementation

Creatine (Cr) supplementation is widely used to enhance HIE performance. In a seminal study, Harris et al. [30] showed that the muscle content of Cr and PCr can be increased by dietary supply of Cr. Oral intake of Cr increases the concentration in blood and muscle, and part of the increased muscle Cr is transformed to PCr—a process catalyzed by Cr kinase. In an average person, Cr supplementation increases total Cr (TCr = Cr + PCr) by about 20 %, with the PCr component accounting for 10 % of the increase [31]. However, there are large differences in the response between subjects, with a more pronounced effect in subjects with low initial muscle TCr content (e.g. vegetarians) and absent in subjects with already high initial contents of TCr, suggesting a ceiling of maximal TCr of 150–160 mmol/kg dm.

It is well documented that Cr supplementation can increase performance during HIE, especially during interval exercise when PCr is the dominating anaerobic source of ATP [32–34]. Cr loading prior to or during a training period can also, due to the ergogenic effect of Cr, increase the training load and thus enhance the training adaptation. This may explain the increased gain in muscle strength when resistance training is combined with Cr loading [35].

### Mechanisms of the Ergogenic Effect of Creatine

The obvious ergogenic mechanism of Cr loading is that the increased muscle content of PCr increases anaerobic capacity. During sustained HIE, PCr contributes about 26 % of the anaerobic capacity (for references, see Sect. 2), and the 10 % increase in PCr would correspond to an increased anaerobic capacity of about 3 % (10 % × 0.26). The reduced catabolism of adenine nucleotides after Cr loading during HIE [34] gives experimental support for the role of Cr in improving energetic status.

PCr–Cr acts as a temporal buffer of ATP, attenuating decreases in cellular ATP during high rates of energy demand. Cr kinase, the enzyme catalyzing bidirectional conversion of Cr–PCr, is localized to structural components close to sites of ATP utilization (myofibrils, sarcoplasmatic reticulum, and sarcolemma) and ATP formation (mitochondria) [36]. This provides the basis for PCr–Cr acting as a spatial buffer of ATP, by which intracellular gradients of ATP–ADP are diminished. Spatial buffering of ATP will reduce increases in ADP at the sites of energy consumption and may be an important factor to prevent contractile failure and fatigue [36, 37]. Increasing PCr by Cr loading will improve both the temporal and the spatial ATP-ADP buffering and explain part of the ergogenic effect.

Another mechanism by which Cr loading can increase performance is related to muscle buffering. PCr-Pi accounts for more than 50 % of the total muscle buffer capacity [23]. It can be calculated that the improved muscle buffering after Cr loading, together with the knowledge that glycolysis accounts for 74 % of anaerobic ATP production (sustained exercise at *V*O_2max_), can increase anaerobic capacity by about 4 % (10 × 0.5 × 0.74). The combined effect of Cr loading on ATP buffering and proton buffering may thus increase anaerobic capacity during sustained exercise by about 6 %. However, due to the heterogenic response between subjects to Cr loading, one would expect that some subjects would benefit more, whereas others would not have any effect at all (non-responders).

An additional function of the PCr–Cr system is in the control of oxidative phosphorylation. Cr kinase is localized to the inner mitochondrial membrane and forms a functional unit with adenine nucleotide translocase [38]. Increases in Cr and ADP stimulate oxidative phosphorylation, whereas PCr has an inhibitory role [39]. Although it is clear that the PCr–Cr couple have a role in controlling aerobic ATP production, at least in oxidative muscle fibers, there is limited evidence that Cr supplementation can improve performance during endurance exercise.

Cr loading has also been shown to increase the rate of force relaxation in human muscle [40]. A faster relaxation would certainly be an advantage during sprints, where the rapid movements require a fast adjustment of muscles between their contraction and relaxation phases.

### Strategies of Creatine Supplementation

A loading regime used in many studies is ingestion of 20 g of Cr monohydrate each day over a period of 5–7 days [30, 31]. The substance should be dissolved in water and distributed in 5 g doses in order to attain sufficient increases in plasma Cr concentration [30]. The loading phase is followed by a period with a lower dose (2–3 g of Cr per day), which is sufficient to maintain the elevated muscle Cr level [31]. The muscle uptake of Cr is largest during the initial loading phase, when about 30 % of the dose administered is stored in muscle tissue and the remaining part excreted as creatinine in the urine [30]. High doses of Cr after a loading phase, where the ceiling level of 150–160 mmol/kg dm has been reached, will be of no use since the surplus of Cr will be excreted in the urine. Muscle uptake of Cr can be enhanced by exercise, and the likely mechanism is increased muscle blood flow and thus exposure of the exercising muscle to elevated plasma Cr [30]. However, muscle Cr uptake is also stimulated by insulin [41], and the stimulating effect of exercise may thus, at least in part, relate to an exercise-induced increase in insulin sensitivity and/or increased exposure of the muscle to insulin. The practical recommendation is that Cr intake should be combined with a substantial amount of carbohydrate and exercise [41].

### Increased Body Weight After Creatine Loading

Cr loading is associated with an increase in body weight of approximately 2 % [42]. Although it has been suggested that Cr loading might stimulate protein synthesis and muscle growth, the evidence for this is limited. It is more likely that the increased body weight after Cr loading is related to increased tissue water content due to the osmotic effect of increased intracellular concentrations of PCr and Cr or to increased glycogen storage [43]. In subjects with otherwise stable body weight, the increased body weight may be used as a rough marker of the effect of Cr loading. The increased body weight will negatively affect performance in running and other sports where body weight influences energy demand, and may therefore reduce the potential ergogenic effects of Cr loading [44].

## Bicarbonate Supplementation

Sodium bicarbonate has been used as an ergogenic aid for many years. Bicarbonate–CO_2_ accounts for more than 90 % of the plasma buffer capacity, and bicarbonate supplementation will increase pH and bicarbonate concentration in blood. The concentration of bicarbonate is much lower in muscle (~10 mmol/L, [45]) than in blood (~25 mmol/L), and the low permeability of the charged bicarbonate ion precludes any immediate effects on the acid-base status of muscle. The rationale for the ergogenic effect of bicarbonate is that the increase in extracellular pH and bicarbonate will enhance the efflux of lactate and H^+^ from muscle [46]. Studies have confirmed that blood lactate is higher after exercise in bicarbonate-supplemented conditions [11]. The increased removal of lactate/H^+^ from the muscle will increase glycolytic ATP formation, thus explaining the ergogenic effect of bicarbonate. Most studies show a documented ergogenic effect after bicarbonate loading during exhaustive exercise lasting 1–7 min [47], which is the period when anaerobic glycolysis will have a major role in energy provision. The effect of bicarbonate on performance is dose dependent, and meta-analyses show that the average increase in performance with doses of 300 mg/kg bm is about 2 % [11]. There is also evidence that the ergogenic effect of bicarbonate is more pronounced during repeated sprints than during sustained exercise [11]. Bicarbonate loading during training may enhance muscle oxidative capacity [48, 49] but has no effect on muscle buffer capacity [49]. Further studies are required to investigate the ergogenic effects of bicarbonate provision over a long-term training period, with special attention paid to the potential negative side effects on electrolyte status.

A major problem that has limited the use of bicarbonate as an ergogenic aid is the gastrointestinal distress with abdominal pain and diarrhea experienced by many individuals [50]. The problems may be reduced by dividing the dose into several small doses combined with ample intake of water [51], or co-ingested with food [50]. Most studies have used acute ingestion of bicarbonate 1–3 h prior to competition, a procedure associated with high risk for gastrointestinal problems. An alternative procedure, which could minimize gastrointestinal distress, is to supplement bicarbonate over several days prior to competition. The homeostatic control of extracellular bicarbonate concentration is a rather slow process controlled by the kidney, and the ergogenic effect of bicarbonate can last up to 2 days after the last dose [52].

## Beta-Alanine Supplementation

Based on the work by Harris and colleagues [53–55], chronic supplementation with β-alanine has gained interest as an ergogenic aid during HIE. The basic idea is that muscle carnosine, which is a natural existing dipeptide of β-alanine and histidine, is an important buffer in skeletal muscle. Carnosine is formed in muscle tissue by synthesis from β-alanine and histidine, and formation of carnosine is limited by the availability of β-alanine [54, 55]. Increasing the plasma concentration of β-alanine by oral intake of β-alanine will thus promote carnosine formation and increase muscle carnosine concentration. The concentration of carnosine in human muscle (vastus lateralis) is about 20 mmol/kg dm, with a twofold higher concentration in fast-twitch than in slow-twitch fibers [55]. The concentration of carnosine is higher in sprinters, rowers, and bodybuilders and is likely explained by the fiber type difference [55]. Carnosine has an equilibrium constant (pKa) of 6.83, making it an ideal intracellular buffer, accounting for about 5–10 % of total muscle buffer capacity. The muscle concentration of carnosine is quite different between species. Species that frequently experience hypoxic periods with high loads of lactic acid, and thus have a great need of H^+^ buffering (e.g. whales, horses, and greyhound dogs), have the highest concentration of muscle histidine containing dipeptides—10–20 times that of an average man [56]. The higher carnosine concentration and buffer capacity in sprinters than in marathoners [57] may, at least in part, be a consequence of a higher proportion of type II fibers.

Oral supplementation with 6 g of β-alanine/d for 10 weeks increased muscle carnosine concentration by 80 % [54]. Under the assumption that carnosine accounts for 8 % [54] of muscle total buffering capacity, and that 74 % of anaerobic capacity is due to anaerobic glycolysis, one would expect that anaerobic capacity would increase by ~4 %. The effect will be less during short periods of exercise, when the contribution from glycolysis to anaerobic ATP synthesis is less.

Several studies have documented that beta-alanine supplementation can improve performance during HIE. Meta-analyses show that supplementation with a total amount of β-alanine of 180 g would result in a median improvement in the outcome of ‘exercise measures’ of 2.9 % [58]. The ergogenic effect was most pronounced during exercise lasting 1–4 min [58], which is the duration when incurred acidosis will be at its peak and of most importance for exercise performance, supporting the proposed ergogenic role of carnosine as a buffer.

To sum up, there is clear evidence that β-alanine supplementation can increase muscle carnosine concentration and thus muscle buffer capacity, but that prolonged periods with large doses are required. The ergogenic effect seems well documented during events lasting 1–4 min, during which lactic acidosis will be most prominent.

## Conclusion

Anaerobic capacity has an important role in HIE performance and can be improved by training and/or nutrition. Training can increase the contracting muscle mass through both hypertrophy (more muscle) or altered fiber/muscle recruitment (more efficient use of existing muscles). An increased contracting muscle mass will increase anaerobic capacity through increased availability of PCr and increased lactate production. Training will not increase muscle PCr content but will increase muscle buffer capacity, which is the first line of defense against lactic acidosis.

Nutritional intervention can add to training-induced improvements in anaerobic capacity. Cr loading will increase anaerobic capacity through both by elevating the muscle PCr content and through improved buffering capacity. Beta-alanine and bicarbonate supplementation can also improve buffering capacity. There is convincing evidence that interventions that increase anaerobic capacity also improve performance during HIE. Furthermore, the ergogenic effects of Cr, beta-alanine, and bicarbonate will enable athletes to increase the training load, and an improved training adaptation may be as important as the direct effects of the supplements.

It should be mentioned that some investigators have argued, based on experiments in isolated rodent muscle or muscle fibers, that lactic acidosis is not related to fatigue or even that lactic acid is an ergogenic drug. However, there is convincing evidence from in vivo studies in exercising humans that dietary interventions that reduce acidosis also improve performance, giving strong support for the idea that lactic acidosis is an important factor in fatigue.

As outlined in this review, there is clear evidence that Cr, bicarbonate, and beta-alanine have ergogenic effects during HIE. However, many commercial products are contaminated with steroid-like chemicals. About 20 % of non-hormonal nutritional supplements contained anabolic androgenic steroids [59]. Although Cr, bicarbonate, and beta-alanine are not classified as illegal substances according to the list of prohibited substances published by the World Anti-Doping Agency (WADA), there is evidence that athletes using these supplements risk inadvertent doping infractions due to contaminated products.
